# Big Data Needs Big Governance: Best Practices From Brain-CODE, the Ontario-Brain Institute’s Neuroinformatics Platform

**DOI:** 10.3389/fgene.2019.00191

**Published:** 2019-03-29

**Authors:** Shannon Lefaivre, Brendan Behan, Anthony Vaccarino, Kenneth Evans, Moyez Dharsee, Tom Gee, Costa Dafnas, Tom Mikkelsen, Elizabeth Theriault

**Affiliations:** ^1^ Ontario Brain Institute, Toronto, ON, Canada; ^2^ Indoc Research, Toronto, ON, Canada; ^3^ Centre for Advanced Computing, Kingston, ON, Canada

**Keywords:** Brain-CODE, governance, ethics, privacy, open data

## Abstract

The Ontario Brain Institute (OBI) has begun to catalyze scientific discovery in the field of neuroscience through its large-scale informatics platform, known as Brain-CODE. The platform supports the capture, storage, federation, sharing, and analysis of different data types across several brain disorders. Underlying the platform is a robust and scalable data governance structure which allows for the flexibility to advance scientific understanding, while protecting the privacy of research participants. Recognizing the value of an open science approach to enabling discovery, the governance structure was designed not only to support collaborative research programs, but also to support open science by making all data open and accessible in the future. OBI’s rigorous approach to data sharing maintains the accessibility of research data for big discoveries without compromising privacy and security. Taking a Privacy by Design approach to both data sharing and development of the platform has allowed OBI to establish some best practices related to large-scale data sharing within Canada. The aim of this report is to highlight these best practices and develop a key open resource which may be referenced during the development of similar open science initiatives.

## Introduction

Data sharing and collaborative research has been widely recognized as a catalyst for scientific discovery (National Institutes of Health, 2003; Wellcome Trust (2010); Canadian Institutes of Health Research, 2013). The Ontario Brain Institute (OBI)^1^ is a provincially funded, not-for-profit research center seeking to maximize the impact of neuroscience and establish Ontario as a world leader in brain research, commercialization, and care (Stuss, 2014; Stuss 2015; Stuss et al., 2015). OBI supports research programs in the areas of neurodegeneration, epilepsy, mood disorders, neurodevelopmental disorders, and cerebral palsy. These programs were created to foster collaborative research and team science by supporting the involvement of multiple institutions in multi-site and multi-modal projects to address key research questions in a harmonized manner. At the time the research programs were created, there were no practical means to facilitate collaboration and ensure standardization across datasets. Disparate databases which existed within institutions lacked the ability to federate different data types within a single research site, across research sites, or across the research programs themselves. To meet the needs of the research programs, an informatics platform, known as Brain-CODE,^2^ was developed to accelerate scientific discovery with the aim of improving the lives of those living with brain disorders (for a technical report on this platform, see Vaccarino et al., 2018).

The collection, sharing, and analysis of personal health information, however, raise a number of governance issues, including agreements between those providing and using the datasets, patient privacy, and the consent process. The key to enabling such collaborations and maintaining long-term research partnerships is a robust and scalable data governance structure which allows for the flexibility to advance scientific understanding, while protecting the privacy of research participants. OBI has developed a governance framework which allows over 40 Canadian research institutions and 600 users to work collaboratively within a centralized data sharing platform. With leading security, privacy policies, and governance infrastructure incorporated into the foundation of Brain-CODE, OBI has been designated a “Privacy by Design” ambassador by the Office of the Information and Privacy Commissioner of Ontario (Cavoukian, 2011). This rigorous approach maintains the accessibility of research data for insightful discoveries without compromising the privacy of research participants. Taking a Privacy by Design approach to both data sharing principles and the development of the platform has allowed OBI to establish some best practices related to large-scale data sharing within Canada. The aim of this report is to highlight these best practices, and develop a key open resource which may be referenced during the development of similar open science initiatives.

## Enabling the Collection, Use, and Disclosure of PHI

The Brain-CODE platform was designed to support curation, sharing, and analysis of multi-modal neuroscience data collected across several brain disorders. Brain-CODE is hosted and managed within the province of Ontario, Canada, and is therefore governed by provincial legislation, the Personal Health Information Protection Act, 2004, S.O. 2004, c.3, Sched A (Personal Health Information Protection Act [PHIPA], 2004a). PHIPA outlines rules associated with the collection, use, and disclosure of personal health information, and is applicable to all health information custodians in Ontario, in addition to organizations which receive personal health information from health information custodians. PHIPA defines health information custodians as persons or organizations who have custody or control of personal health information as a result of or in connection with performing the person’s or organization’s powers or duties (e.g., hospitals, long-term care facilities, etc.). Under PHIPA (Personal Health Information Protection Act, 2004b), OBI operates as an electronic service provider in accordance with s. 10(4), and provides electronic means to support health information custodians in the collection, use, disclosure, retention, and disposal of health data. As an electronic service provider, OBI has the ability to support collaborative research by providing a secure platform onto which data custodians can transfer and interrogate research data.

KEY CONCEPT 1 **Electronic service provider**

A person who supplies services for the purpose of enabling a health information custodian to use electronic means to collect, use, modify, disclose, retain, or dispose of personal health information and who is not an agent of a health information custodian, as contemplated in Ontario Regulation 329/04 of PHIPA.

Another means by which OBI can support collaboration through Brain-CODE is by operating as a health information network provider, in accordance with s. 6(2) of PHIPA (Personal Health Information Protection Act, 2004b), by providing electronic means for two or more health information custodians to disclose personal health information to one another. A health information network provider differs from an electronic service provider in that the services provided are primarily for the purpose of disclosing personal health information. The utility of Brain-CODE lies not just within the ability to disclose personal health information, but also to allow health information custodians to use personal health information and work collaboratively. While the Brain-CODE infrastructure is capable of supporting the activities of a health information network provider, there has not yet been a use case to date where these services have been required. Thus, OBI functions mainly as an electronic service provider.

KEY CONCEPT 2 **Health information network provider**

A person who provides services to two or more health information custodians where the services are provided primarily to custodians to enable them to use electronic means to disclose personal health information to one another, whether or not the person is an agent of any of the custodians, as contemplated in Ontario Regulation 329/04 of PHIPA.

## Streamlining Data Transfer Agreements

An important distinction to make is that as an electronic service provider, OBI is not considered a health information custodian, with respect to PHIPA. This classification has an impact on data governance policies, given that OBI does not have custody or control of the data that are transferred to Brain-CODE. To allow for the transfer of data from the data custodians to the electronic service provider (Brain-CODE), a robust data transfer agreement is required. To this end, OBI has developed a standardized Participation Agreement which is executed with all institutions before data are transferred to Brain-CODE. Some key features of this agreement include an outline of how data will be transferred, stored, and disclosed, including the associated privacy obligations of the respective parties, as well as terms binding the institution to OBI’s governance and security policies. By allowing institutions to review and provide feedback on the terms of the agreement before execution, OBI can provide confidence that the privacy and security requirements/standards of the institution are met by Brain-CODE.

KEY CONCEPT 3 **Participation agreement**

A robust data transfer agreement which is executed with all institutions before data are transferred to Brain-CODE. Some key features of this agreement include an outline of how data will be transferred, stored, and disclosed, including the associated privacy obligations of the respective parties, as well as terms binding the institution to OBI’s governance and security policies.

One of the major advantages of the standardized Participation Agreements, executed between each institution and the electronic service provider, lies within the administrative efficiencies that are created. Individual data sharing agreements between each institution involved in multi-site studies are not required, which greatly reduces the number of agreements requiring institutional review. Additionally, the Participation Agreement is structured such that the overall principles are agreed upon up front, and study-specific details are captured in a Study Description Schedule that is appended to the agreement.

KEY CONCEPT 4 **Study description schedule**

Schedule of the Participation Agreement that includes information specific to each individual research project that will utilize Brain-CODE. This includes details such as study protocol, REB approval, consent forms, description of datasets to be transferred, institutional conditions, and a list of investigators directly involved as collaborators in the study who will have access to the data in an identifiable form.

**Figure 1:**
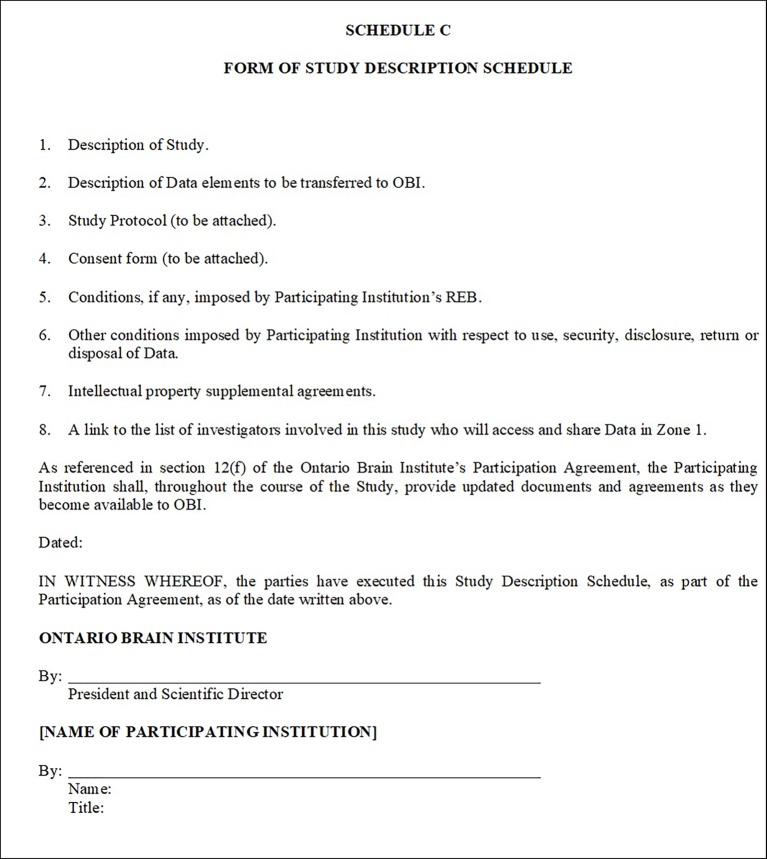
Study Description Schedule of the OBI Informatics Participation Agreement.

The Study Description Schedule (Figure 1) includes information specific to each individual research project that will utilize Brain-CODE. This includes details such as study protocol, REB approval, consent forms, description of datasets to be transferred, institutional conditions, and a list of investigators directly involved as collaborators in the study who will have access to the data in an identifiable form. While the list of investigators is important to track, this list often requires updates on a regular basis due to the nature of research. To support evolving research teams, and to remediate the need for continual amendments to the Study Description Schedule, an online link to a list of investigators was created for each research program. The creation of this link allows for the list of collaborators to be dynamic, much like the research environment, and provides for the most updated information within agreements. This same link is used within the consent language so that study participants can also be aware of changes to the research team. A Study Description Schedule is executed for each research study, with the associated permissions and restrictions tracked centrally to ensure compliance.

The overall structure of the Participation Agreement and Study Description Schedules can be likened to a Master Services Agreement model, whereby Statements of Work are appended to represent the activities and deliverables and the overall agreement represents the terms agreed upon between parties. Each time a new study from an institution will utilize Brain-CODE, it does not require the execution of a new Participation Agreement, but rather a new Study Description Schedule is appended to the overall agreement. This streamlined approach allows the addition of new studies to be expedited through institutional contracts offices, reducing the number of administrative barriers to initiate collaborative research projects.

## Standardizing Participant Consent to Share Data

OBI acts as an electronic service provider and therefore does not have custody or control of personal health information. Brain-CODE operates based upon informed participant consent. There are several benefits to obtaining informed consent for data sharing initiatives, including the opportunity for participants to influence future uses of their data, as well as increased transparency and understanding of the proposed initiative. There are also challenges associated with obtaining informed consent for a collaborative project which requires research ethics board approval across several research institutions, and therefore the use of several consent form versions. In an effort to harmonize and streamline the process, OBI has created standardized informed consent language that is used across all research projects that plan to use Brain-CODE.^3^ This language was developed in collaboration with research ethics board chairs from the network of participating institutions and the Information and Privacy Commissioner of Ontario in 2015. The language is incorporated within existing study informed consent forms to describe in clear lay language how data will be transferred to Brain-CODE, in what form, and to explain the nature of open data initiatives. The standardized informed consent language has been widely adopted across more than 100 research projects, and well received by the research participants.

KEY CONCEPT 5 **Standardized informed consent**

Language which is incorporated within existing study informed consent forms to describe in clear terms how data will be transferred to Brain-CODE and in what form, and the nature of open data initiatives.

### Managing Participant Consent and Ethics Permissions

Given that Brain-CODE operates based upon informed participant consent, institutional REB approvals and associated informed consents govern which data can be collected, uploaded, de-identified, and shared on Brain-CODE. Although OBI has implemented standardized consent language, there are cases where the language is modified, and it becomes important to track this information in an easily accessible way. For all studies, this information is tracked in a centralized Brain-CODE Ethics Tracking Database, which contains information on the sensitivity of datasets and sharing permissions. Each record within the Ethics Tracking Database represents a single informed consent form and the associated REB approval. Within each record, the data permissions and restrictions are captured in a standardized format, and reviewed with the data custodians to mitigate any potential discrepancy between the consent language and REB approval. Some examples of information captured in the database include what personal health information is approved for transfer (e.g., full date of birth, month and year, age only), whether the participant has consented to de-identification for third-party data sharing, who has access to the identifiable dataset, and any other restrictions for the use of the data. Thus, OBI does not simply track whether or not informed consent was received for data transfer to Brain-CODE, but rather determines at a granular level the nature of the obtained consent. In addition to tracking data permissions at the study level, the information in the Ethics Tracking Database is also linked to each participant *via* a custom-built Web-based Subject Registry application which allows the tracking and management of data permissions on a participant-by-participant basis. This allows for an in-depth view of data permissions, and provides guidance to ensure compliance to research ethics.

KEY CONCEPT 6 **Ethics tracking database**

Centralized database which contains standardized information on the sensitivity of datasets and sharing permissions where each record represents a single informed consent form and the associated research ethics board approval.

**Figure 2:**
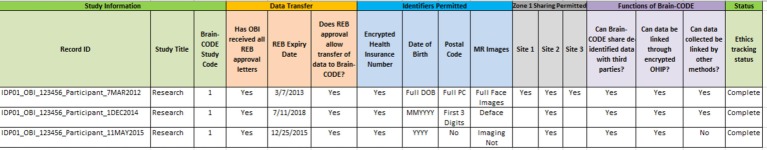
Sample Ethics Restrictions Report, an output of Brain-CODE’s Ethics Tracking Database.

Ethics Restrictions Reports are outputs from the Ethics Tracking Database, which are produced for each study to provide an overview of the dataset permissions (Figure 2). These reports are used as a tool for both the researchers and OBI service providers. Before data enter the platform, the Ethics Restrictions Report is used as a guide to ensure that data collection forms (such as electronic case report forms) are in compliance with ethics restrictions. While it is the responsibility of the researchers at the participating institutions to ensure that data which do not have approval for transfer to Brain-CODE are not uploaded, OBI has the opportunity to assist with compliance. The Ethics Restrictions Report informs field level validations to ensure that only data which have been consented and REB approved can be transferred. For example, if the approved format for date of birth is month and year, a check would be done to ensure that field level restrictions are in place to ensure that day of birth is not captured. The report is also utilized during project creation, as well as user account creation and management processes. Before projects are created in Brain-CODE, the report is consulted to ensure that all project documentation has been received, including Participation Agreement, Study Description Schedule, and REB approval. The report is also used to manage Brain-CODE accounts to ensure that project access is restricted to authorized users.

KEY CONCEPT 7 **Ethics restrictions reports**

An output from the Ethics Tracking Database produced for each study to provide an overview of dataset permissions.

## A Tiered Approach to Data Access

**Figure 3:**
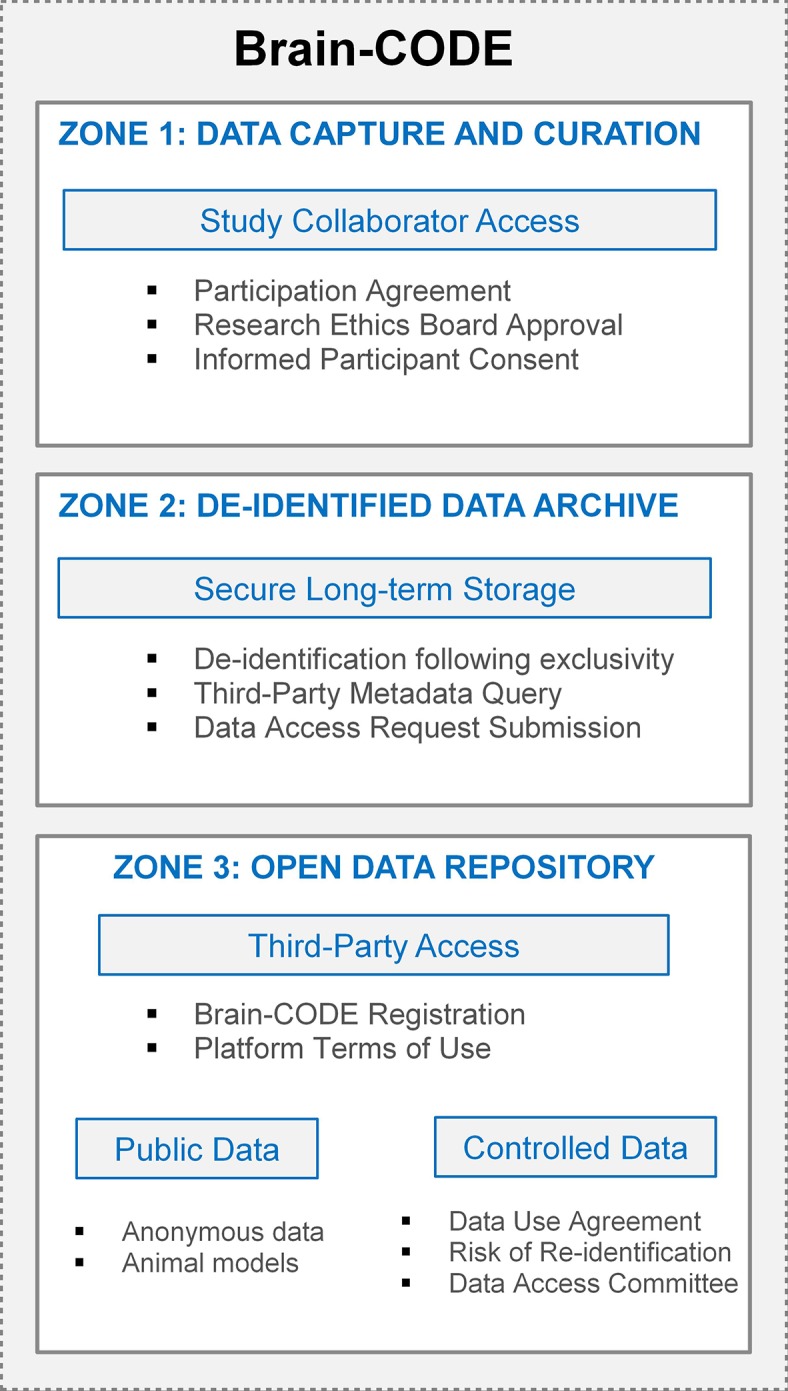
Brain-CODE’s secure three-zone infrastructure.

Although open access and data sharing are fundamental concepts to an open science approach to scientific discovery, individual-level participant data require adherence to the individual agreements, participant consent, and the over-riding legal and ethical standards governing the collection and sharing of those data. To address this, a tiered approach to data organization in Brain-CODE has been implemented that enables granular access permissions to allow sensitive data to be transferred to the platform, while ensuring only authorized users have access to datasets. OBI has developed a highly secure, three-zone infrastructure for Brain-CODE which provides functional separation of sensitive data for controlled access (Figure 3).

Zone 1 supports the electronic capture and transfer of raw data to Brain-CODE, and provides tools to facilitate the management, curation, analysis, and sharing of the dataset. Within Zone 1, it is only direct study collaborators, as per the list of investigators referenced in the informed consent and Study Description Schedule, who could have access to the data. Each of OBI’s funded research programs has a data administrator who is responsible for submitting account access requests to Brain-CODE; these requests are cross-referenced with the Ethics Restrictions Report prior to granting access to ensure research ethics compliance. Given the focus on open science, a condition of Brain-CODE use is that all datasets be available for open access in the future. Once curated, datasets typically undergo a one-year period of exclusivity where direct collaborators have exclusive access to the datasets in Zone 1 prior to third-party access. The purpose of implementing an exclusivity period is to provide data sharing opportunities among collaborators while reassuring researchers that their data will remain confidential, secure, and appropriately protected from release during their exploration of the data.

Once the exclusivity period has elapsed, the datasets are de-identified and transferred into Zone 2, a secure long-term storage area. Provisions within the Participation Agreement grant OBI a non-exclusive license to use the datasets, in a de-identified form, indefinitely to support open data initiatives. While datasets in Zone 2 are not yet accessible to external researchers, they are integrated with the Brain-CODE federation system to allow metadata query. External researchers interface with these metadata data through an interactive data visualization dashboard to select and submit data access requests for datasets they require for analyses.

Zone 3 is a virtual workspace for external researchers to access and analyze open datasets. The number one priority when sharing data is participant privacy, therefore data sharing on Brain-CODE is facilitated in accordance with participant informed consent and REB approval. Within Zone 3, there are two levels of data access which are defined by the sensitivity of the dataset: Public releases and Controlled releases. Datasets which have not previously contained PHI (e.g., animal models, anonymous data) are classified as Public, while datasets which previously contained PHI and have since been de-identified are classified as Controlled. Access to any open data requires registration of a Brain-CODE user account through a user portal. Registration is a mechanism to validate users, provide an opportunity for users to review and agree to the Brain-CODE terms of use, while at the same time collecting metrics to track the impact of open data.

### Data Access Committee

Access to Controlled data is further managed by a data access request process and reviewed through a two-tier process by the Brain-CODE Data Access Committee and Brain-CODE Informatics Steering Committee (Shabani et al., 2015). The Data Access Committee is composed of representative researchers from each of OBI’s funded Integrated Discovery Programs, neuroinformatics experts, and OBI staff. Allowing for researchers involved in the collection of the data to provide feedback on data access requests is important to build trust among the research community, and assurances against potential misuse or misinterpretation of dataset. The data access request process requires external researchers to briefly describe the proposed study and the potential impacts the findings may have on furthering our understanding of brain disorders and improving brain health. External researchers are required to sign a data use agreement, providing assurance that data will be used only for the purposes described in the data access request, as well as to provide proof of REB approval when required by their institution. Prior to disclosure to third parties, all data are stripped of identifying information to the extent possible using advanced de-identification tools and, in addition, an analysis of the risk of re-identification is performed. These tools are designed to both assess and consequently minimize the risk of re-identification of datasets. The data access request processes have been streamlined to allow for requests to be processed within 14 days. External researchers who have been granted access to data in Zone 3 are contacted annually for updates related to data use, including discoveries and publications. OBI encourages the return of results to the Brain-CODE platform, including protocols, algorithms, and code developed to benefit the broader scientific community. The tiered access model of OBI’s governance structure demonstrates the ability to foster data sharing and collaboration, without compromising participant privacy.

KEY CONCEPT 8 **Data access committee**

Committee which informs the development of data access and sharing policies, data exclusivity, data acknowledgment, and ethics requirements, and makes recommendations to the Informatics Steering Committee regarding the release of data to third parties.

## Data De-Identification

A key privacy principle which underlies OBI’s policies is that of collection limitation (Canadian Standards Association, 1996; Cavoukian, 2011). During the initial design and conception of Brain-CODE, it was envisioned that the platform would only host de-identified data to minimize privacy risks. Following consultation with OBI’s funded research programs it was discovered that in order for the platform to have a higher level of utility for direct collaborators involved in a project, some identifiers, such as date of birth, were critical to support analyses. As an electronic service provider, OBI can support the transfer of personal health information that has been collected and is required for the purposes of the research study and analyses. Where institutional research ethics boards have approved such information and informed consent has been obtained, only researchers involved in the study have access to the dataset, including the identifiers, in a secure Zone 1 environment.

Direct identifiers which provide an explicit link to a study participant, such as name, are removed at source before datasets are transferred to Brain-CODE. Any identifiers which are not required to support analyses, and therefore do not have research ethics board approval for transfer, are not uploaded to the platform. Datasets are coded in Zone 1, by replacing participant names or other identifying information with a unique subject identifier, to allow researchers to track participants within a study. The subject identifiers are required to adhere to a standardized naming convention that is used across all datasets in Brain-CODE to ensure uniqueness.

Beyond access in Zone 1 by direct study collaborators, datasets are not accessible in an identifiable format. All structured clinical data elements are irreversibly anonymized, whereby a process removes the association between the identifying data and the study participant (International Organization for Standardization, 2017). Methods utilized to de-identify indirect identifiers include suppression, generalization, and sub-sampling, with the aim to maintain the highest level of data utility while also protecting study participant privacy (El Emam, 2013). High-resolution magnetic resonance images may be processed using a defacing pipeline to remove facial features from the image without compromising the integrity of brain regions required for analysis (Bischoff-Grethe et al., 2007). In addition, for all magnetic resonance images, any identifying information captured in the image header is removed at source before transfer. There are challenges related to the de-identification of genomics datasets, due to the rapid evolution of methods and technologies leading to a lack of best practices and standards within this data modality.

Clinical components of the anonymized datasets undergo a risk of re-identification assessment to ensure the risk level is minimized, and within the acceptable range for the given release context. The risk assessment takes into account both the risks associated with the dataset and the risks associated with the data release context (El Emam, 2013). The data set risk is minimized by employing the amount of de-identification individually required by the dataset to reduce the risk of participant re-identification. The de-identification methods described above are utilized to reduce the dataset to a risk level that is acceptable by OBI before release. Analyses by external researchers are governed by data use agreements, and whenever possible analyses will be restricted to the secure Brain-CODE environment. These additional administrative controls are taken into account during the clinical data risk assessment, as this allows for flexibility in the anonymization methods used on the dataset to maintain the highest level of data utility. Taken together, the outcome of the risk assessment produces a dataset which balances protecting participant privacy by applying a level of de-identification necessary to minimize risk while also optimizing dataset utility.

## Privacy Preserving Data Linkages

Data linkages provide the opportunity to maximize the impact of research data by allowing for existing datasets to be leveraged for enriched analyses. Within Brain-CODE, there are two types of data linkages which can be facilitated: participant-level linkage and cohort-level linkage. Participant-level linkage occurs when participants in Brain-CODE are known to exist in an external database, and a common identifier is leveraged to combine the datasets and correctly associate a participant’s data across the datasets. Cohort-level linkage does not associate individual participants within disparate datasets, but rather combines datasets based upon probabilistic matching of similar cohort characteristics (i.e., participants with major depressive disorder).

Participant-level linkage can only occur in Zone 1, where there are sufficient identifiers to allow for such a linkage. In the province of Ontario, a key identifier is the Ontario Health Insurance Plan (OHIP) number, which is unique for each of the 13.6 million residents in the province. The OHIP number is a direct identifier which provides a link to health administrative databases, and thus is a highly sensitive data element. Collecting the OHIP number from research participants allows for research data to be linked with participant-level health administrative data, creating opportunities to track the development of diagnoses, treatment responses, and other long-term health outcomes. To facilitate this, OBI partnered with Indoc Research and Dr. Khaled El Emam to create a privacy preserving protocol which allows for the capture of encrypted OHIP numbers. Once encrypted, the OHIP numbers are no longer considered personal health information. OHIP numbers are encrypted using the Subject Registry software running within a Web browser on-site at the institutions, prior to any data being transferred to Brain-CODE (Vaccarino et al., 2018). The software employs a method of homomorphic encryption (El Emam et al., 2012) which utilizes a public key to ensure the original identifier does not leave the research site, and only ciphertext is transferred to Brain-CODE. The private key for decryption is inaccessible to OBI and is securely maintained by a trusted third party. The encryption algorithm allows for mathematical computations to be applied to the encrypted data to perform comparisons which can securely integrate datasets, without requiring decryption of the original data. Linked datasets are accessible for analyses within a secure, access-controlled environment where download of sensitive data is prohibited. This functionality not only allows the potential to link with external databases, but also for de-duplication of participants within Brain-CODE that have participated in multiple research studies. This methodology could be applied to other direct identifiers, such as medical record numbers, to facilitate similar participant-level linkages.

Cohort-level linkage does not require individual identifiers, and therefore can occur in Zone 1; however, they most commonly involve de-identified datasets in Zone 3. Given the purpose of de-identification is to prevent individual participant re-identification and linkage, cohort-level association is a privacy-preserving way to increase dataset sample size for analysis. Such linkages are governed by federation agreements between OBI and external data providers, in addition to research ethics board approval. A risk of re-identification assessment is performed before a linked dataset is accessible, to ensure the combining of cohorts has not affected participant privacy.

## Collaborative Policy Development

Developing the Brain-CODE informatics platform and the associated governance policies to support neuroscience researchers from over 40 institutions across Canada would not have been possible in isolation. Having policies and procedures informed by key stakeholder groups allows for the assurance that policies will meet institutional requirements for privacy and security, creating a trusted relationship and a streamlined path to adoption across the institutions. Brain-CODE policies and infrastructure development have received guidance from two international advisory committees: the Brain-CODE Analytics Advisory Committee and the Brain-CODE Advisory Committee. Both committees regularly meet with OBI to advise on the direction and progress of Brain-CODE development to ensure the platform continues to meet international best standards related to data management, sharing, and analysis. Within OBI, the Brain-CODE Informatics Steering Committee provides routine oversight of the development, implementation, and operations of the platform. The Data Access Committee is consulted during the development of data access and sharing policies, data exclusivity, acknowledgement, and ethics requirements, and makes recommendations to the Informatics Steering Committee regarding the release of data to external researchers. The Information Security Committee provides recommendations regarding an appropriate information security framework for the Brain-CODE platform and works with the OBI to identify, implement, and maintain privacy standards for all data in Brain-CODE.

Key stakeholders outside of the OBI governance structure include research ethics boards and privacy officers from participating research institutions, as well as the Information and Privacy Commissioner of Ontario. It is beneficial to engage these stakeholders in discussions during the early phases of large-scale data sharing initiatives, to ensure that platform infrastructure, policies, and processes can support specific institutional privacy and security standards, while also aligning with legislative requirements. Continual engagement of these groups to communicate updates and receive feedback on new initiatives is valuable and creates a culture of trust among participating institutions in utilizing Brain-CODE as an electronic service provider.

## Actionable Recommendations and Conclusions

To date, there are over 17,000 study participants in Brain-CODE, across OBI’s five supported brain disorder areas: neurodevelopmental disorders,^4^ cerebral palsy,^5^ epilepsy,^6^ mood disorders,^7^ and neurodegenerative disorders.^8^ With the amount of data increasing daily, and new institutions expressing interest in using the platform, or adopting the Brain-CODE model of data sharing, the OBI Informatics Governance Policy has set a high standard for the governance of data sharing in the neuroscience field. OBI has developed a framework which allows for the flexibility to meet institutional and participant privacy and security requirements while promoting open science and data sharing initiatives. Participation Agreements with research institutions allow for the transfer of data to Brain-CODE and streamlined addition of new studies, whereby OBI functions as an electronic service provider. Standardized informed consent language creates transparency and understanding of the initiative and provides participants with an opportunity to influence future uses of their data. Centralized tracking of data permissions informs robust data access processes, which are complemented by a secure three-zone structure to ensure functional separation of sensitive datasets. By creating the governance and supporting infrastructure to allow for privacy-preserving data linkages, OBI is able to maximize the time invested by research participants to contribute their data.

KEY CONCEPT 9 **Three zone structure**

Tiered approach to data organization which provides functional separation of sensitive data for controlled access, enabling granular access permissions to allow more sensitive data to be transferred to the platform, while ensuring only access is restricted to authorized users.

The key recommendation to new data sharing initiatives is to prioritize the organization and implementation of a robust governance structure at the outset of a project. It is challenging to retrospectively enforce a governance structure once a project has started, whereas it is easier to build a project to align with a governance structure from the start. Adopting a Privacy by Design approach to data sharing creates an environment which is open for enabling opportunities as the project progresses. The engagement of key stakeholders early in the process is important, as it provides an opportunity for organizations involved to ensure their privacy and security standards are met. It also builds a relationship on a foundation of trust, which streamlines adoption of the policies among organizations. Given the nature of open data initiatives, it is difficult to predict all future uses of data; therefore, it is important to create a structure which allows for flexibility, while maintaining the focus on participant privacy.

The governance structure of an electronic service provider and the associated administrative processes, such as obtaining informed consent and executing institutional data transfer agreements, are perceived by some to create barriers to scientific discovery. OBI believes strongly in the informed consent model of open data initiatives and in empowering participants to have control over their data; rather than creating barriers, this model of governance creates opportunities to enable fresh approaches and partnerships. An open science mentality that adheres to the highest standards of privacy and security ensures that the use of research data is maximized, and gives hope to those living with brain disorders, and respect to those who have participated in research studies.

## Author Contributions

SL wrote the first draft of the paper and prepared the manuscript. All authors contributed to the development of Brain-CODE policies and commented on/revised the manuscript.

### Conflict of Interest Statement

The authors declare that the research was conducted in the absence of any commercial or financial relationships that could be construed as a potential conflict of interest.

## Footnotes

^1^www.braininstitute.ca

^2^www.braincode.ca

^3^Brain-CODE standardized informed consent language is available online at: https://www.braincode.ca/content/getting-started and in supplementary material

^4^https://pond-network.ca/

^5^http://cpnet.canchild.ca/

^6^http://eplink.ca/

^7^http://www.canbind.ca/

^8^http://ondri.ca/
