# Supplementary material for: Big Data Needs Big Governance: Best Practices From Brain-CODE, the Ontario-Brain Institute’s Neuroinformatics Platform
Source: Front Genet. 2019 Mar 29;10:191. doi: 10.3389/fgene.2019.00191 (PMC6450217; doi:10.3389/fgene.2019.00191)
Supplement: Supplementary file 1 [file Table_1.DOCX]

**Generic Consent Language for Brain-CODE**

**Which data will be collected and why?**

These data are being collected as part of an Ontario collaborative study on [*ID Program*], and will be used in conjunction with data collected from persons with the same and different conditions. This will allow researchers to study the causes of the disorder, improve diagnoses, and develop treatments and interventions. In addition to the data that will be collected from [*study intervention*], we will also be collecting your health card number and other demographic information like [*insert details*]. Your encrypted health card number or [*other data we collect in this study*] may be used to link with data stored in independent databases, such as OHIP, or other databases in a secure environment.

By providing consent to this study, you are agreeing that the data collected during this study will be stored electronically in a database developed and maintained by the Ontario Brain Institute (OBI). This database is known as Brain-CODE, and it will be stored in reputable facilities with high level security protocols. Brain-CODE will allow researchers to use the data collected in these collaborative studies to perform research and learn more about how [*disease*] affects the brain.

**What will happen to my data?**

Brain-CODE is an open-access database. This means that researchers and organizations outside this study can request access to data that have been stripped of information that can identify you. Your health card number will be encrypted upon entry into Brain-CODE and will remain encrypted at all times.

**How will my information be kept confidential?**

We wish to assure you that your privacy is very important to us. When you join the study, you will be given an ID number. Researchers will use this ID number to organize your data, instead of your name or other information that can identify you directly. Any data collected for study purposes that could potentially identify you will be stored in a highly secure manner and never be released or disclosed in a form that could identify you. We will use tools that remove identifying information using the most advanced tools available to minimize the risk of identifying you from the information we collect or release. This process will be applied to all data, including any new data that have been linked through other databases, such as OHIP.

[*Organization*] and OBI have entered into legal agreements to protect your data, and to set out the purposes for which these data will be collected, used, stored and disclosed. Steps have been taken to make sure your data are safe and the risk of identifying you is minimized. The OBI will continue to monitor these safeguards as new technologies evolve in order to limit any new risks to privacy.

**Who will have access to my information, and what will they see?**

Data collected through this study and stored in Brain-CODE will be available to researchers in this study. A current list of these researchers and organizations can be found at: [*Email* [*governance@braincode.ca*](mailto:governance@braincode.ca) *for the program specific link*].

Data from this study that have had identifying information removed using the most advanced tools available may be shared with local, national and international researchers and organizations that are not part of this study. This open approach is being used by researchers internationally to better understand disease. Access to data by outside researchers or organizations will require a detailed plan for the use of the data, and approval from a research ethics board, as described in OBI’s Data Sharing Policy <http://www.braininstitute.ca/Brain-CODE-governance>. These researchers or organizations will be required to enter into an agreement with OBI that clearly states the safeguards that will be in place to protect those data, and the purposes for which these data may be collected, used, stored and disclosed.

OBI may take some of your data, combine them with data from many other people, and make them available to enhance the public’s awareness of research. We will use tools to remove identifying information from these combined data sets, making the risk of identifying you minimal.

**If I decide later on that I no longer want to be part of this study, what happens?**

You can withdraw from the study at any point. No new data will be collected or linked to other data from that point on. Upon your request, any data that have not been processed to remove identifying information will be destroyed. However, we are not able to remove any data that have already been analyzed, processed to remove identifying information or linked with other data for placement in Brain-CODE. To withdraw from the study, contact [*Site PI; email; phone*].

If you have any questions, please ask [*appropriate person at site*]. Further information about Brain-CODE is available at <http://www.braininstitute.ca/Brain-CODE-governance>.

Any of the information in this letter can be sent to you by mail, upon your request.
